# Minimum Inhibitory Concentrations of Anidulafungin as a Potential Predictor of Biocide Susceptibility of Clade 1 *Candidozyma* (*Candida) auris Isolates*

**DOI:** 10.1002/mbo3.70093

**Published:** 2025-10-21

**Authors:** Sidre Erganis, Ayse Seyer, Mubarak Taiwo Mustapha, Meliz Yuvali, Furkan Martli, Sena Algin, Sema Turan Uzuntas, Beyza Yavuz, Alper Dogan, Esra Kılıc, Fusun Kirca, Abdullahi Garba Usman, Elif Ayca Sahin, Cagrı Ergin, Bedia Dinc, Dilber Uzun Ozsahin, Ayse Kalkanci

**Affiliations:** ^1^ Department of Microbiology Faculty of Medicine, Gazi University Ankara Türkiye; ^2^ DESAM Research Institute Near East University Nicosia Cyprus; ^3^ Operational Research Center in Healthcare Near East University Nicosia Turkey; ^4^ Department of Biostatistics Faculty of Medicine, Near East University Nicosia Cyprus; ^5^ Clinics of Microbiology, Bilkent City Hospital Ankara Türkiye; ^6^ Research Center of Experimental Health Science Near East University Nicosia Cyprus; ^7^ Department of Microbiology Faculty of Medicine, Pamukkale University Denizli Türkiye; ^8^ Department of Medical Diagnostic Imaging University of Sharjah, College of Health Sciences Sharjah United Arab Emirates

**Keywords:** antifungal, artificial intelligence, biocide, *Candidozyma (Candida) auris*, machine learning, random forest regressor, virulence

## Abstract

*Candidozyma (Candida) auris* is a multidrug‐resistant yeast capable of causing persistent outbreaks in healthcare settings. Its reduced susceptibility to both antifungals and biocides poses a direct threat to infection control and hospital hygiene, as biocides remain the cornerstone of surface disinfection and outbreak containment. This study aimed to construct a decision tree to predict biocide susceptibility and identify key predictive parameters using machine learning. Virulence factors were evaluated in 55 *C. auris* strains isolated from a state hospital in Türkiye that were identified by MALDI‐TOF and verified by DNA sequencing. CLSI guidelines were followed in determining the antifungal MICs. The *z*‐score method was used to standardize and numerically code phenotypic data. The Random Forest Regressor was used to analyze feature importance. Resistance thresholds were defined as triclosan ≥ 0.5 µg/mL, benzalkonium ≥ 150 µg/mL, chlorhexidine ≥ 1.0 µg/mL, and chlorine ≥ 0.03 µg/mL. As all strains were benzalkonium‐resistant, these data were excluded. Anidulafungin MIC was the strongest predictor of biocide sensitivity, followed by amphotericin B, flucytosine, and isavuconazole, while other virulence factors showed little or no value. This proof‐of‐concept study demonstrates, for the first time, that a decision tree model trained on antifungal MIC profiles can predict the susceptibility of *C. auris* strains to triclosan, chlorine, and chlorhexidine. Although biocide susceptibility testing was performed to establish reference thresholds, the final predictive framework relied solely on anidulafungin MIC values, suggesting that such models may reduce the need for routine biocide testing in future surveillance studies.

## Graphical abstract

Our study shows that a decision tree trained on antifungal MIC profiles, especially anidulafungin, can predict how susceptible Candida auris is to triclosan, chlorine, and chlorhexidine. This shows a possible way to cut down on routine biocide testing in infection control surveillance.

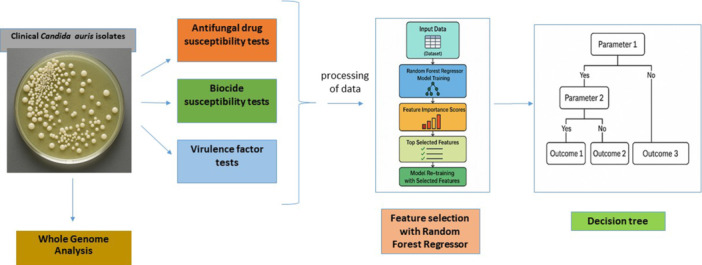

## 1 Introduction

*Candidozyma (Candida) auris* is a multidrug‐resistant (MDR) pathogen listed in the prioritized fungi by World Health Organization (WHO) published in 2022. Rapid spread across continents has raised concerns about its epidemiology, treatment, and control measures after its initially isolated in 2009 from an external auditory canal of a Japanese patient. It has emerged as a significant global health threat due to its ability to cause healthcare‐associated infections (HAIs) with high morbidity and mortality rates (Eix and Nett 2025).

Genetic analyses of *C. auris* revealed that this yeast consists of six clades, each geographically distinct, but each is capable of simultaneous and independent emergence. The genetic clades demonstrate phylogeographic mixing, although certain regions, such as South America (Clade IV), shows stronger microbial fitness (Hayes 2024). *C. auris* is uniquely capable of surviving on dry and moist surfaces for extended periods, contributing to nosocomial transmission. Its biofilm‐forming capacity, halotolerance, and thermotolerance distinguish it from other *Candida* species (Nwachukwu et al. 2023). A proposed hypothesis links *C. auris* emergence to climate change, suggesting that global warming facilitated thermal adaptation in an environmental ancestor, enabling its pathogenicity and global dissemination. This underscores the One Health approach, emphasizing the interconnectedness of human, animal, and ecosystem health in understanding and mitigating *C. auris* (Casadevall et al. 2021; Zhai et al. 2021; Arora et al. 2021).

Conventional diagnostic methods often misidentify *C. auris*, necessitating advanced techniques like mass spectrometry (MALDI‐TOF) and DNA sequencing. Misdiagnosis delays appropriate treatment, leading to poor outcomes (Fasciana et al. 2020). Current therapeutic options are limited by the pathogen's MDR nature, with echinocandins being the first‐line treatment. However, number of resistant isolates to all major antifungal classes are rising (Ganeshkumar et al. 2024).

In Turkiye, limited data exist regarding the prevalence of *C. auris* (Erkose Genc et al. 2024; Bölükbaşı et al. 2021). Despite the global spread of *C. auris*, Türkiye lacks systematic national surveillance, and published data are limited to isolated case reports and small‐scale studies. This creates a critical blind spot for regional epidemiology and infection control since Türkiye occupies a geographically strategic position bridging Europe, Asia, and the Middle East. This highlights the need for effective control of *C. auris* which requires a multidisciplinary approach. Infection prevention measures, such as enhanced hygiene protocols, contact tracing, and patient isolation, are critical in healthcare settings. Despite its growing prevalence, our knowledge regarding its susceptibility to biocides remains limited (Erganis et al. 2024). The complexity of performing reliable biocide susceptibility testing further compounds this knowledge gap. Standardized protocols for biocide susceptibility testing are scarce, and results are often inconsistent across laboratories. This variability arises from differences in testing methodologies, concentration ranges, and environmental factors. Additionally, *C. auris* biofilm formation and resistance mechanisms further complicate susceptibility assessments (Horton and Nett 2020).

Antifungal susceptibility testing, on the other hand, is more standardized and commonly used in clinical settings. The potential relationship between antifungal and biocide resistance patterns in *C. auris* remains an intriguing and underexplored area. To our knowledge, no prior studies have systematically examined whether antifungal MIC profiles can predict biocide susceptibility in *C. auris*. This study, therefore, represents the first attempt to test this hypothesis using machine learning. Understanding this connection could pave the way for innovative predictive models. We hypothesize that antifungal susceptibility profiles could serve as proxies for estimating biocide susceptibility. Machine learning algorithms could be instrumental in analyzing complex datasets to identify correlations between these susceptibility patterns.

## 2 Materials and Methods

The study was approved by the Ethics Committee of Gazi University Faculty of Medicine (Date: February 6, 2023, No: 115).

### 2.1 Phenotypic and Genotypic Identification of the Isolates

A total of 55 clinical *C. auris* isolates were sent from Ankara Bilkent City Hospital to Gazi University Faculty of Medicine, Department of Medical Microbiology laboratory. Additionally, a quality control strain (CDC MBL *Candida auris* B11903) was included in the study as the 56th strain. All isolates were identified by MALDI‐TOF MS (Vitek‐MS, bioMerieux, France). Genomic confirmation was made for eight selected srains by “Next generation sequencing” (NGS) of fungal “Internal Transcribe Sequences (ITS)” gene located in rRNA. Digestion buffer (250 mM NaCl, 100 mM EDTA, pH = 8, 1% SDS, 8 M guanidine thiocyanate) was used for DNA isolation and purified using phenol‐chloroform‐isoamyl alcohol (25:24:1). The amount of DNA was checked by spectrophotometrically (NanoDrop). For WGS, samples were barcoded using Oxford Nanopore protocols, and sequence was performed on a MinION Mk1C device. Libraries were prepared using Ligation Sequencing Kit (SQK‐LSK109) and Native Barcoding Kit (EXP‐NBD104‐114). Amplification products were validated on agarose gel (~1450 bp) and purified. Final DNA libraries were loaded onto a Spot‐On Flow Cell (FLO‐MIN106D) for sequencing using the Mk1C device with MinKNOW™ software. Post‐sequencing, fastq reads were processed, trimmed using Trimmomatic, and filtered. Reads shorter than 2000 bp were excluded from the analysis. Raw data were converted to fastq format and processed using Guppy software for base‐calling and demultiplexing. A custom Python‐based workflow incorporating BLAST algorithms was used for taxonomic classification. Qiime2 Platform was used for OTU‐based analyses, including alpha/beta diversity, PCA, and PCoA. Whole genome analysis (WGS) was performed for clade classification. The sequencing reads were processed and analyzed using Geneious Prime 2023.2.1 for optimal sequence configuration. For species identification, alignment was performed using local BLAST version 2.12.0 + . Genome data were deposited in the “The GenBank” (https://www.ncbi.nlm.nih.gov) and accession numbers were obtained including CDC strain *C. auris* (B11903). Strict contamination control procedures were applied throughout DNA extraction and sequencing. Negative extraction controls (blank samples) and no‐template PCR controls were included in each batch to monitor contamination. Library preparation and sequencing were conducted in physically separated clean areas with filtered pipette tips and UV‐sterilized workspaces. To ensure reproducibility, representative isolates were sequenced in duplicate (technical replicates) and yielded consistent results across runs. Sequencing reads were further screened for potential contaminants using BLAST against the NCBI database, and no non‐*Candidozyma* sequences were detected.

### 2.2 Antifungal Drug Susceptibility Tests

“Clinical and Laboratory Standards Institute (CLSI)” M27‐A2 reference microdilution method was performed for *C. auris* strains. Drug concentrations were as 16‐0.03 µg/ml for amphotericin B, 64–0.125 µg/mL for voriconazole, 128‐0.25 µg/mL for fluconazole, 16–0.03 µg/mL for itraconazole, posaconazole, caspofungin, isavuconazole, anidulafungin and flucytosine. Minimum inhibitory concentration (MIC) values were noted. *Candida albicans* ATCC 10231 was the quality control. The evaluation of the susceptibility was made according to “Centers for Disease Control and Prevention” (CDC) breakpoints for *C. auris*. The MIC values over 2 µg/mL for amphotericin B, 32 µg/mL for fluconazole, 2 µg/mL for caspofungin, and 4 µg/mL for micafungin and anidulafungin were accepted as being resistance breakpoints (https://www.cdc.gov/candida-auris/hcp/laboratories/antifungal-susceptibility-testing.html).

### 2.3 Susceptibility Tests of Biocides by Broth Microdilution

Sensitivity tests for biocides were performed using microdilution method, which has been adapted from CLSI M27‐A2 reference microdilution method. Biocide concentration were as follows; triclosan (TRC) (0.0078–8 mg/L), chlorine (CHLOR) (0.0078–8 mg/L), chlorhexidine (CHX) (0.0625–32 mg/L), and benzalkonium chloride (BNZ) (0.0625–128 mg/L). The thresholds for determining biocide susceptibility, which are crucial as they are based on established clinical and microbiological guidelines, indicate the MIC value below which a strain is considered susceptible to a biocide. For biocides, susceptibility thresholds (e.g., triclosan ≥ 0.5 µg/mL, chlorhexidine ≥ 1.0 µg/mL, chlorine ≥ 0.03 µg/mL, benzalkonium ≥ 150 µg/mL) were adopted from Morrissey et al. (2014) and subsequent studies on biocide susceptibility testing in clinically relevant microorganisms (Morrissey et al. 2014). These cut‐offs have been used in prior biocide‐resistance evaluations and provide a standardized basis for comparison. While inter‐laboratory comparisons were not feasible in this study, the methodology strictly followed CLSI reference standards (M27‐A2), ensuring comparability with published susceptibility data.

### 2.4 Virulence Factors

Biofilms of *C. auris* strains were formed in 96‐well flat‐bottomed microplates and washed twice with 200 µL sterile distilled water. 50 µl MTT (5 mg/mL) was added to the washed biofilms. The aligners were incubated in the dark at 37°C for 2 h at 80 rpm. The biofilm‐forming abilities of the isolates were graded. Those with an optical density (OD) value (570 nm) between twice the OD value of the negative control and the OD value of the negative control were considered weak biofilm (1 + ), those with an OD value between twice the OD value of the negative control and four times the OD value of the negative control were considered moderate biofilm (2 + +), and those with an OD value more than four times the OD value of the negative control were considered strong biofilm (3 + + +) (Angiolella et al. 2024).

Other than biofilm ability, enzymatic activities such as hemolytic, protease, phospholipase, casein, and esterase activities were also evalutated. Hemolytic activity test was performed using Sabouraud Dextrose Agar (SDA) containing 7% sheep blood. At the end of 72 h incubation the formation of hemolysis zones (alpha, beta, or gamma hemolysis) around the isolates will be observed. Detection of SAP activity evaluated by using agar medium containing bovine serum albumin (SSAA). After 96 h of incubation, the development of a clear lysis zone around the isolates indicated SAP activity. Investigation of phospholipase activity tested with the production of enzymes that degrade phospholipids. Egg yolk agar (YSA) was used. The formation of a precipitation zone around the isolates demonstrated phospholipase activity. Esterase activity was investigated by using Tween 80 agar. The presence of a precipitation zone around the isolates confirmed esterase activity. Casein activity evaluated by milk agar contains 2% milk. After 72 h of incubation, the presence of a clear zone around the isolates indicated the hydrolysis of casein and confirmed caseinase activity. Qualitative phenotypic factors were converted into numerical values to facilitate ML analysis. The following conversions were applied: biofilm, SAP factors: “Negative” = 0, “+” = 1, “++” = 2, “+++” = 3. For hemolytic activity, phospholipase, caseinase, and esterase activities “Negative” = 0 and “Positive” = 1 (Çuhadar et al. 2018).

All enzymatic activity assays were performed in triplicate, and results were reported as the mean of three independent experiments. Negative controls (media without inoculum) and positive controls (reference *C. albicans* ATCC 10231 strain for SAP, phospholipase, and hemolysin activity) were included in each batch of assays to ensure reliability and reproducibility of the measurements.

### 2.5 Feature Scaling

Feature scaling, a significant step in our research, ensures that all features contribute equally to the analysis by normalizing the range of independent variables or features of data. In our study, the StandardScaler scales the features before handling class imbalance and splitting the data, ensuring that the decision tree classifier receives appropriately scaled data for more effective learning and accurate predictions. These steps ensured that all features contributed equally to the model and that the scales of the features did not disproportionately influence the results. Phenotypic/genotypic factors were standardized to have a mean of 0 and a standard deviation of 1 using the *z*‐score formula: x_std = (*x* − *μ*)/*σ.*

To minimize bias related to a small sample size, fivefold cross‐validation was applied during model training. Performance metrics (accuracy, precision, recall, F1 score) were averaged across folds to ensure stability of results. Additionally, bootstrapping with 1000 resamples was conducted to estimate variability in feature importance rankings, providing confidence intervals for key predictors.

### 2.6 Feature Selection

Feature selection is a critical process in the development of ML models. It aims to identify and select the most relevant features from the data set for model training.

We used embedded methods to incorporate feature selection as part of the model training process, utilizing algorithms with built‐in feature selection capabilities, such as Lasso (L1 regularization), decision trees, and tree‐based ensemble methods like “Random Forest Regressor” and “Gradient Boosting Machines.” While filter‐based approaches (e.g., univariate correlation, chi‐square) and wrapper methods (e.g., recursive feature elimination) were not systematically applied in this study, exploratory analyses indicated consistent ranking of antifungal MIC variables across different approaches. Embedded methods were prioritized due to their ability to capture nonlinear interactions and reduce overfitting risk in relatively small datasets.

In our study, feature importance is evaluated using the decision tree classifier, which inherently ranks features based on their contribution to reducing impurity, allowing insights into which features are most significant for predicting each target variable. This step helps understand the underlying data structure and ensures that the model focuses on the most impactful features. The “Random Forest Regressor” was trained using the preprocessed data set, and the number of trees (n_estimators) was set to 100. Other hyperparameters were set to ensure optimal performance. These hyperparameters include the maximum depth of the trees (max_depth) set to 5, the minimum number of samples required to split an internal node (min_samples_split) set to 2, the minimum number of samples needed to be at a leaf node (min_samples_leaf) set to 1, and the seed for reproducibility (random_state) set to 42. Relevant features were selected based on their potential impact on predicting biocide susceptibility. The features included phenotypic and genotypic factors (Biofilm, SAP factor, casein activity, phospholipase activity, esterase activity, and hemolytic activity (alpha, beta, gamma)) and MIC values of antifungal drugs (amphotericin, voriconazole, fluconazole, itraconazole, posaconazole, caspofungin, isavuconazole, anidulafungin, and flucytosine). These features were chosen for predicting biocide susceptibility. Although virulence factor data (biofilm formation, SAP, hemolysin, phospholipase, esterase, caseinase) were initially included in the feature set, Random Forest analysis consistently assigned negligible importance scores (< 0.05). As a result, these variables were excluded from the final predictive decision tree models.

Before *z*‐score standardization, all continuous variables were examined for outliers and distributional skewness. Outliers beyond three standard deviations from the mean were inspected and retained unless attributable to technical error, to avoid artificially constraining biological variation. MIC data were approximately log‐normal, and log2 transformation was applied before *z*‐score scaling to reduce skewness. Although z‐score normalization assumes linearity, we verified this by visual inspection of histograms and Q‐Q plots, confirming that transformed distributions approximated normality.

### 2.7 Decision Tree

A decision tree model is a versatile and interpretable ML algorithm for classification and regression tasks. We formed by recursively splitting the data set into subsets based on the value of input features, forming a tree‐like structure where each internal node represents a decision on a feature, each branch represents the outcome of the decision, and each leaf node represents a class label or a continuous value. This hierarchical, rule‐based approach allowed decision trees to capture complex interactions between antifungal and virulence and their corresponding biocide outcomes. The resulting model visualized, making it straightforward to understand how predictions are made and which features are most influential. It is followed by evaluation using cross‐validation and metrics like confusion matrix and classification report to validate its effectiveness on different target variables. The grid search is employed to tune the hyperparameters of a decision tree classifier, such as the criterion for splitting, maximum depth, minimum samples for splitting, and minimum samples per leaf. The data set was randomly divided into training and test subsets for model evaluation. Confusion matrices were generated solely from the test subset to prevent information leakage and to ensure unbiased performance assessment. As a result, the distribution of resistant and susceptible isolates in the matrices does not necessarily reflect the biological frequencies in the complete data set.

## 3 Results

### 3.1 Feature Importance

**Figure 1:**
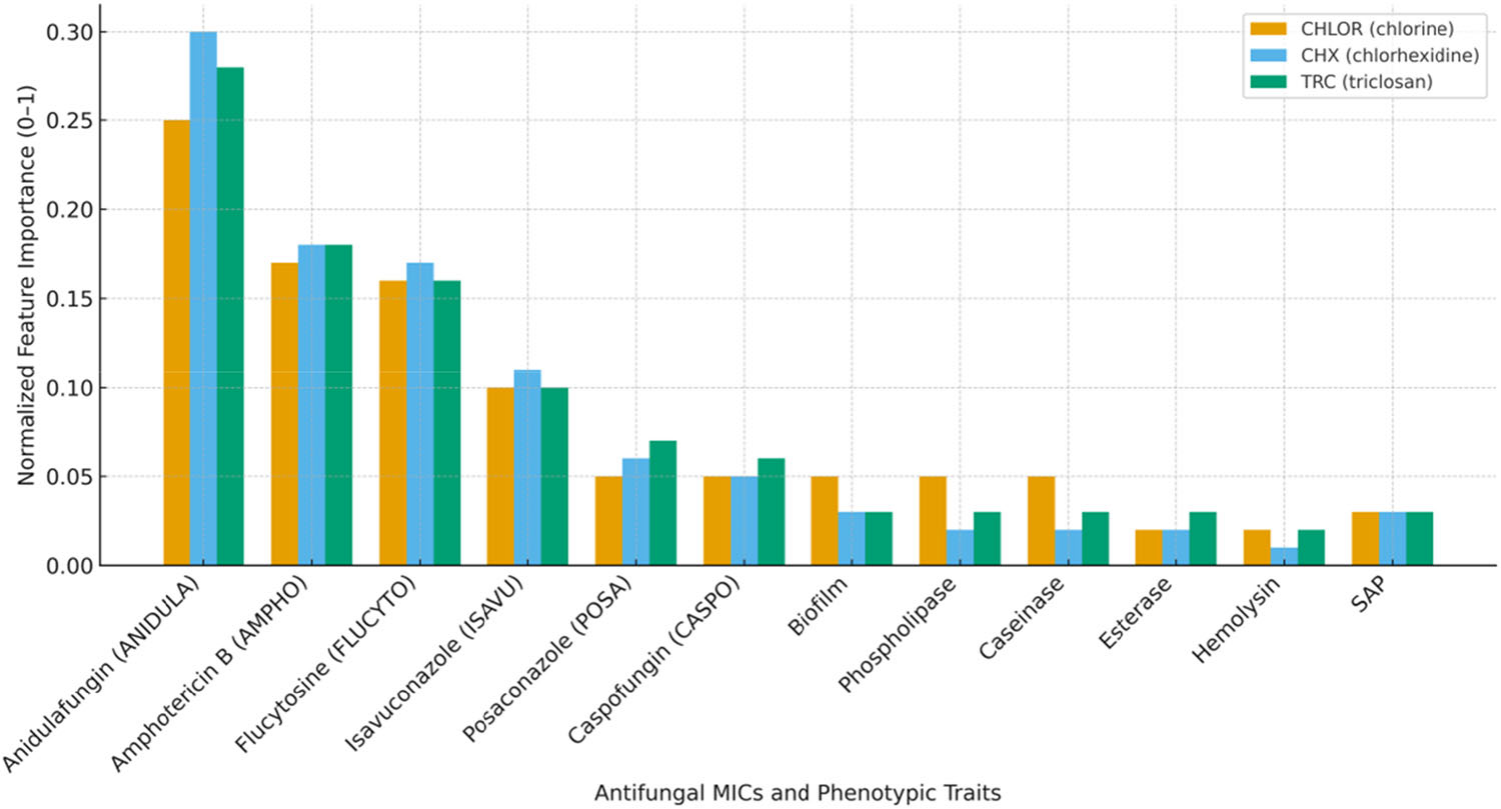
Feature importance analysis of antifungal MICs and virulence factors for predicting biocide susceptibility in *Candidozyma (Candida) auris*. Colored bars represent the relative contribution of each variable to the prediction of resistance/susceptibility for three biocides: chlorine (CHLOR, orange), chlorhexidine (CHX, blue), and triclosan (TRC, green). Feature importance scores were calculated using the Random Forest Regressor based on mean decrease in impurity, averaged across 100 trees and fivefold cross‐validation. Scores were then normalized to allow comparison across models. Higher values indicate stronger predictive influence of the feature in the corresponding biocide‐specific model.

Virulence factor assays demonstrated consistent expression of several traits among the isolates. All 55 isolates exhibited α‐hemolysis, biofilm formation, and secretory aspartyl proteinase (SAP) activity. Biofilm formation was robust across the cohort, with 95% of isolates showing strong slime production (+++ by MTT assay). Esterase activity was detected in 41 isolates (74.5%), whereas only 2 isolates (3.6%) were positive for caseinase activity. None of the isolates demonstrated phospholipase activity. While these data confirmed the widespread presence of multiple virulence attributes, feature importance analysis indicated that these traits contributed negligibly to the predictive modeling of biocide susceptibility. In this study, we utilized the “Random Forest Regressor” to determine the importance of features in predicting biocide susceptibility in *Candidozyma auris* strains. We prefer the “Random Forest Regressor” over the “Decision Tree Regressor” because it provides more stable, reliable, and comprehensive estimates by averaging the results from multiple trees. This reduces the risks of overfitting and sensitivity to data variability, resulting in a more accurate representation of feature importance, as indicated in Figure 1.

The feature importance analysis using the “Random Forest Regressor” highlighted the key predictors of biocide susceptibility in Clade 1 *C. auris* strains. ANIDULA stands out as the most influential feature, with an importance score of approximately 0.27, indicating its significant impact on the model's predictions. This is closely followed by AMPHO and FLUCYTO, with importance scores of around 0.18 and 0.17, respectively, underscoring their crucial roles in determining biocide susceptibility. ISAVU also plays a notable role, with an importance score of 0.11, contributing significantly to the model's output. Other features, such as CASPO and POSA, show moderate importance, suggesting they add predictive value but are secondary to the top features. The phenotypic factors and additional antifungals have lower importance, indicating a lesser impact on the model's predictions. These high‐importance features, particularly ANIDULA, AMPHO, and FLUCYTO, are essential for accurately distinguishing between susceptible and resistant strains, driving the major decisions in the “Random Forest” model. Understanding the importance of these features helps identify key factors influencing biocide resistance, guiding further research an‐drd informing treatment strategies for *C. auris* infections.

### 3.2 The Decision Tree

The decision tree splits the data based on the MIC values of various antifungal drugs. Each decision node refines the prediction by considering additional features, ultimately leading to a leaf node that provides the final predicted MIC values for the biocides.

**Figure 2:**
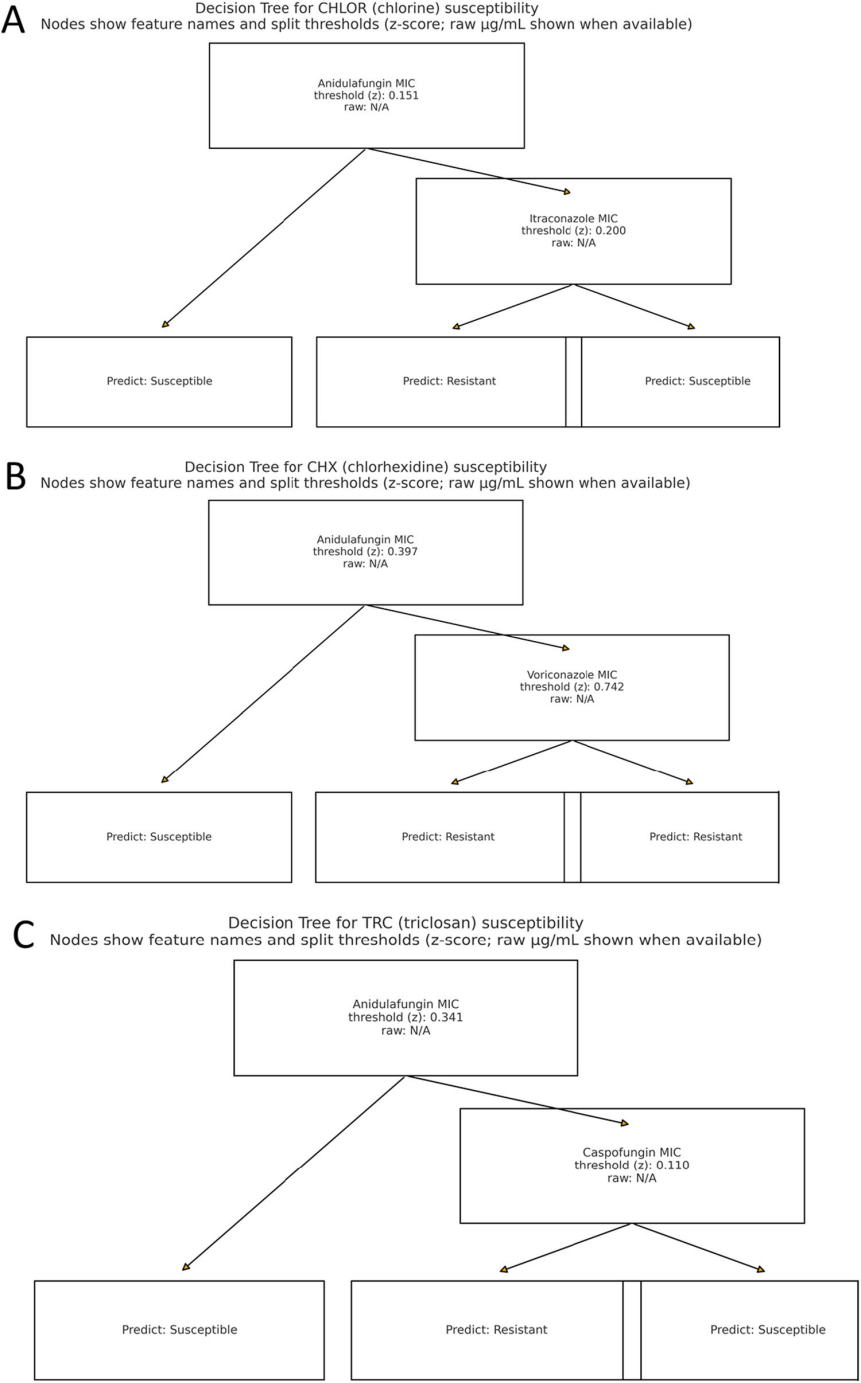
Across all three decision trees (A, B, and C) predicting resistance to CHLOR (split at 0.151, CHX (split at 0.397), and TRC (split at 0.341), anidulafungin (ANIDULA) MIC consistently emerged as the most influential predictor, even when not selected as the root node. Its high discriminative power highlights its central role in estimating biocide susceptibility in C. auris strains, supporting its use as a surrogate marker in the absence of direct biocide testing.

Figure 2 shows the decision trees for three target variables: CHLOR, CHX, and TRC. All 55 clinical isolates and the reference strain demonstrated resistance to benzalkonium chloride at concentrations ≥ 150 µg/mL. As universal resistance precluded meaningful tree modeling, BNZ data were excluded from subsequent predictive analyses. Nevertheless, this consistent phenotype underscores the unique tolerance of *C. auris* to quaternary ammonium compounds.

Each tree shows the hierarchical structure of decisions made by the classifier based on the feature values to classify samples. For the CHLOR target, the tree begins with the feature ANIDULA, split at 0.151. This node divides the samples into two groups, with a Gini impurity of 0.497, indicating a relatively mixed set. On the left, all samples are classified as class 0, while the right side further splits based on the feature ITRA, resulting in another decision node. The final splits lead to pure nodes with low Gini impurity, indicating clear classification decisions. In the CHX tree, the first decision node also uses the ANIDULA feature, splitting at 0.397. This initial split again results in two branches. The right branch, determined by the VORI feature with a split at 0.742, leads to pure nodes with low Gini impurity. The left branch classifies all samples as class 0, while the right side achieves perfect classification with class 1. The TRC tree starts with ANIDULA at a split value of 0.341. This initial decision separates the data into two branches. The left branch immediately classifies all samples as class 0 with zero impurity. The right branch splits on the CASPO feature at 0.11, resulting in low impurity nodes, accurately distinguishing between the classes.

The decision trees demonstrate effective feature selection and splitting criteria, leading to accurate classification with minimal impurity at the terminal nodes. The dominant feature across all trees is ANIDULA, indicating its strong influence on the classification outcomes for these target variables.

### 3.3 Model Performance

The primary goal of this study was to predict *C. auris*'s biocide susceptibility using the MIC values of several antifungal drugs. Based on their responses to these antifungals, the decision tree model was employed to determine which biocides *C. auris* strains might be resistant to or susceptible to. The reference strain and all 55 isolates exhibited susceptibility to chlor; just one isolate demonstrated resistance to triclosan, while three isolates were resistant to chlorhexidine. The results are summarized in the performance metrics in Table 1 and the confusion matrices for the three target biocides: CHLOR, CHX, and TRC.

**Table 1:** Performance evaluation metrics.

Biocide	Class	Definition	Precision (%)	Recall (%)	F1 Score (%)	Accuracy (%)
CHLOR	0	Susceptible	100	71	83	83
CHLOR	1	Resistant	71	100	83	
CHX	0	Susceptible	100	100	100	100
CHX	1	Resistant	100	100	100	
TRC	0	Susceptible	100	94	97	97
TRC	1	Resistant	93	100	96	

**Figure 3:**
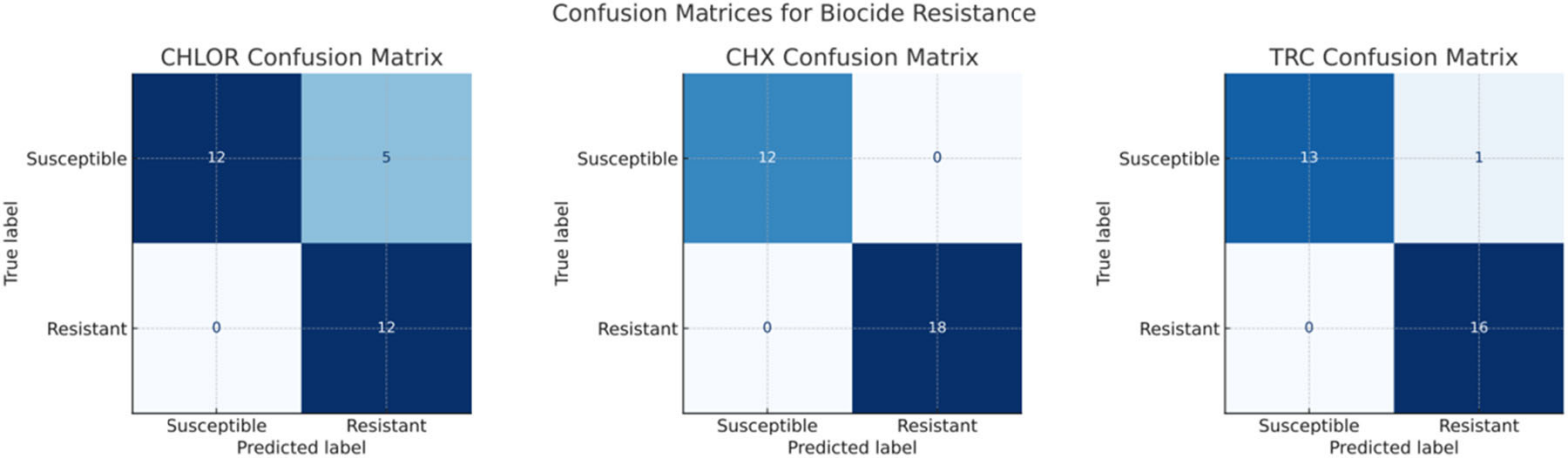
Confusion matrix for biocide susceptibility predicting resistance to chlorine (CHLOR), chlorhexidine (CHX), and triclosan (TRC) using decision tree models. Each matrix shows the distribution of actual versus predicted susceptibility categories.

The confusion matrices for each target biocide, as shown in Figure 3, reveal the number of correct and incorrect predictions made by the model. For CHLOR, there were 12 true positives, 12 true negatives, 5 false positives, and no false negatives. For CHX, the model achieved perfect classification with 18 true positives, 12 true negatives, and no false positives or false negatives. For TRC, there were 16 true positives, 13 true negatives, 1 false positive, and no false negatives.

The high precision and recall values for both CHX and TRC and relatively high values for CHLOR indicate that the decision tree model is highly effective in predicting the biocide susceptibility of *C. auris*, as shown in Table 1. The perfect CHX classification and near‐perfect TRC suggest that the model can reliably identify resistant or susceptible strains to these biocides. This is critical for clinical decision‐making, as it enables healthcare professionals to choose the most effective biocides for treating infections caused by *C. auris*, known for its multidrug resistance.

For CHLOR, the slightly lower precision for the resistant class (71%) suggests room for improvement. However, the recall of 100% for the resistant class indicates that the model is very good at identifying all resistant strains, even if it occasionally misclassifies some susceptible strains as resistant. This conservative approach may be beneficial in a clinical setting to avoid using ineffective biocides.

To reduce the risk of overfitting inherent to decision tree classifiers, we limited tree depth (max_depth = 5) and applied fivefold cross‐validation during model training. Additionally, Random Forest and Gradient Boosting ensemble methods were explored to compare the stability of feature importance rankings. Although these ensembles improved exploratory approachness, decision tree models were reported here due to their interpretability and ease of clinical application.

## 4 Discussion

*C. auris* represents a formidable challenge to global health systems, characterized by its MDR profile, virulence, and propensity for rapid dissemination. Addressing this threat requires coordinated efforts in surveillance, research, and the implementation of innovative diagnostic and therapeutic approaches. The integration of One Health principles and the exploration of advanced technologies like nanomedicine may pave the way for more effective management of this emerging fungal pathogen. Recent analyses show that up to 91% of isolates demonstrate fluconazole resistance, with substantial resistance to other antifungals depending on geographic clades (Chow et al. 2020; Garcia‐Bustos et al. 2023).

In addition to antifungal resistance, *C. auris* demonstrates high tolerance to common disinfectants, particularly quaternary ammonium compounds. This resistance is a major factor in its persistence in healthcare environments, where it colonizes surfaces and medical devices. Biofilm formation on abiotic surfaces further complicates biocide effect. We have limited data about the activites and the detection of resistance to biocides among *C. auris* strains. Biocide susceptibility testing is a complex issue requires standardized protocols. Our results indicated that using a decision tree model to predict biocide susceptibility based on antifungal MIC values provides a exploratory approach and interpretable method for guiding treatment strategies. The encouraging performance and balanced performance metrics underscore the model's potential utility in clinical diagnostics, helping to combat the challenges posed by multidrug‐resistant *C. auris* and improve patient outcomes (Du Toit 2023).

There are limited number of studies that exemplify the role of artificial intelligence (AI), particularly decision trees, in analyzing microbial resistance. In the first study, machine learning and imputation techniques were used to evaluate human norovirus genotype susceptibility to sodium hypochlorite. Genotype‐specific resistance patterns were identified using generalized linear models, random forests, and gradient boosting. Decision trees effectively highlighted genotype interactions with disinfectants, optimizing disinfection strategies (Hamilton et al. 2024).

The second study analyzed *Staphylococcus aureus* resistance to antibiotics and biocides using decision trees and clustering. This large‐scale analysis (1,632 isolates) revealed a correlation between biocide susceptibility and multidrug resistance. The decision trees identified CHX and BNZ MICs as key predictors for antibiotic susceptibility. These findings emphasize AI's utility in uncovering complex relationships in resistance phenotypes (Coelho et al. 2013).

Another study evaluates fluconazole breakpoints for *Candidozyma* infections using data mining algorithms like CART decision trees, comparing methodologies from CLSI and EUCAST. Despite slight variations in MIC thresholds, dose/MIC ratios, and clinical outcomes show strong alignment, validating EUCAST's breakpoints. CART consistently outperformed other classifiers, identifying MIC ≤ 4 mg/L and dose/MIC ≥ 75 as optimal predictors of therapeutic success. Analysis combined data sets from various cohorts, yielding a sensitivity of 90% and specificity of 88%. These findings highlight the utility of data mining techniques in refining antifungal breakpoints and emphasize the need for consensus between global standards to improve clinical decision‐making. The reviewed studies highlight diverse applications of ML in predicting AMR and improving diagnostic and treatment strategies (Cuesta et al. 2010).

Our study confirmed that *C. auris* isolates consistently express key virulence factors, particularly biofilm formation, hemolytic activity, and SAP activity, aligning with prior reports that emphasize the pathogenic potential of this species. The predominance of strong biofilm production (95% of isolates) highlights the well‐recognized environmental persistence of *C. auris*. Despite this, virulence traits did not emerge as significant predictors of biocide susceptibility in our decision tree models. This suggests that while such factors are crucial for host colonization and persistence, biocide tolerance may be more closely tied to intrinsic cellular mechanisms such as cell wall integrity, efflux activity, and oxidative stress responses. By explicitly reporting these findings, we underline that virulence data were systematically collected and analyzed, but ultimately excluded from the final predictive framework due to their limited contribution.

In our study, the random forest regressor analysis identified anidulafungin (ANIDULA) MIC value as the most critical predictor of biocide susceptibility in *C. auris* strains, with an importance score of approximately 0.27. This data‐driven result suggests that anidulafungin possesses greater discriminatory capacity than other antifungals for predicting biocide susceptibility. Mechanistically, as an echinocandin targeting β‐1,3‐glucan synthase, anidulafungin disrupts fungal cell wall integrity, which may also influence cell permeability and stress responses relevant to biocidal action. Taken together, both empirical and biological considerations support the use of anidulafungin MIC as a surrogate predictor in our model. Amphotericin B (0.18) and flucytosine (0.17) MIC values were also highly influential, followed by isavuconazole (0.11). Caspofungin and posaconazole showed moderate importance, while phenotypic factors and other antifungal drugs had minimal impact on the model's predictions. Interestingly, phenotypic virulence traits such as biofilm formation, SAP, hemolysin, and phospholipase activity showed negligible predictive value in our machine learning models. The exclusion of virulence factor data from the final model was driven by their low predictive value, not by methodological shortcomings. All assays were performed with replicates and appropriate controls, ensuring data reliability. However, the lack of predictive contribution indicates that phenotypic virulence traits may not be directly associated with biocide susceptibility in *C. auris*. This finding is noteworthy, as it suggests that antifungal susceptibility profiles provide more exploratory approach predictive signals than virulence phenotypes. Future studies with larger datasets could further explore whether subtle interactions exist between virulence and biocide tolerance that were not captured in our cohort. This outcome contradicts our initial hypothesis that virulence factors might correlate with biocide susceptibility, given their known role in environmental persistence and pathogenicity. The discrepancy likely reflects fundamental differences in resistance mechanisms: while virulence factors enhance colonization and immune evasion, biocide resistance appears more tightly linked to cell wall integrity, stress‐response pathways, and efflux mechanisms. From a methodological perspective, it is also possible that the relatively small data set limited the ability of the model to capture subtle interactions between virulence traits and biocide tolerance. Therefore, our findings suggest that antifungal susceptibility profiles are stronger and more consistent predictors of biocide resistance than phenotypic virulence traits, although larger‐scale studies are warranted to confirm or refute this observation.

Decision trees constructed for three biocides demonstrated that ANIDULA was consistently the primary splitting feature, highlighting its dominant role in determining biocide susceptibility. Each tree accurately classified resistant and susceptible strains with low impurity levels. Although our decision tree models demonstrated encouraging performance and recall, particularly for CHX and TRC, these findings should be interpreted with caution. The predictive power observed here is based solely on internal cross‐validation of a single‐institution data set, without external validation. As such, the performance metrics may overestimate real‐world generalizability. For chlorine, the model showed slightly lower precision for the resistant class (71%), but with a recall of 100%, effectively identifying all resistant strains. The reduced precision of the CHLOR prediction model may stem from several factors. First, the number of resistant isolates for CHLOR was relatively limited, leading to class imbalance and reduced discriminatory power. Second, chlorine's mode of action‐primarily oxidative damage‐is broad and may not correlate as tightly with antifungal MIC profiles as biocides like CHX or TRC, which have more specific membrane‐active mechanisms. Finally, variability in MIC testing for chlorine, which is chemically unstable and influenced by environmental factors, may have introduced additional noise. Although our model maintained high recall (100%) for resistant strains, indicating strong sensitivity in detecting resistance, the lower precision underscores the need for larger, balanced datasets and potentially alternative modeling approaches to improve predictive performance for chlorine.

The universal resistance to benzalkonium chloride observed in our isolates is striking. Quaternary ammonium compounds (QACs), including benzalkonium chloride, are among the most widely used disinfectants in hospital settings. Previous reports have suggested that *C. auris* can persist on surfaces despite QAC‐based cleaning protocols, contributing to its environmental resilience and outbreak potential (Erganis et al. 2024; Du Toit 2023). Our finding of complete BNZ resistance across all isolates highlights the limited efficacy of QACs for *C. auris* decontamination and aligns with infection control recommendations to avoid relying solely on these agents. This observation warrants further investigation into the molecular mechanisms underlying QAC resistance and supports the prioritization of alternative disinfectants, such as chlorine‐ or alcohol‐based formulations, in hospital hygiene protocols. This conservative approach reduces the risk of misclassifying resistant strains as susceptible, ensuring safer clinical applications. These findings indicate that using antifungal MIC values to predict biocide susceptibility is an effective and exploratory approach. The encouraging performance and reliability of the decision tree model underscore its potential utility in clinical diagnostics and infection control strategies, particularly for managing multidrug‐resistant *C. auris*.

Our results suggest that antifungal susceptibility profiles, particularly anidulafungin MIC values, can serve as surrogate predictors of biocide susceptibility. One possible biological explanation is that echinocandins target β‐1,3‐glucan synthase, directly affecting fungal cell wall integrity. Alterations in this pathway may modify cell wall architecture and surface permeability, thereby influencing the penetration or activity of amphipathic biocides such as chlorhexidine and chlorine. In addition, exposure to echinocandins is known to trigger compensatory stress responses, including chitin upregulation and activation of calcineurin signaling. These same adaptive mechanisms may overlap with pathways that confer tolerance to oxidative and membrane‐active biocides. By contrast, virulence factors such as biofilm formation or SAP activity, while relevant for persistence and host interaction, did not align with biocide susceptibility in our data set, underscoring that biocide resistance may be more tightly linked to core cell wall and stress response pathways than to classical virulence traits.

Antimicrobial resistance (AMR) continues to be a pressing global health crisis, and recent advancements in ML offer transformative potential in predicting resistance and optimizing treatment strategies. Ardila et al. emphasize the integration of WGS with ML to predict AMR in critical pathogens, showcasing Random Forest and XGBoost models as top performers with high predictive accuracy (Ardila et al. 2024). Similarly, Wang et al. employ “Random Forest Regressor” to forecast MICs of *Salmonella*, highlighting the value of WGS data in enhancing the precision of surveillance efforts (Wang et al. 2023). The use of biocides and its impact on AMR development is another critical aspect explored by Wesgate et al. who develop a predictive protocol to assess risks associated with biocide exposure (Wesgate et al. 2016). Their findings reveal that exposure to certain biocides, such as TRC, significantly increases the risk of cross‐resistance in bacteria like *S. aureus* and *Escherichia coli*. The role of ML in analyzing biocide‐induced resistance trends could be pivotal in formulating data‐driven policies. Comprehensive databases documenting biocide usage and its microbial impacts are crucial for effective intervention strategies. Explainable AI (XAI) methods, as demonstrated by Cavallaro et al. (2023) address the critical need for transparency and trust in ML applications for AMR. By employing gradient‐boosted decision trees alongside Shapley values, they elucidate the factors influencing resistance predictions, bridging the gap between AI tools and clinical decision‐making. This interpretability is vital for fostering trust among healthcare providers and ensuring patient safety. Furthermore, their approach demonstrates a significant reduction in mismatched antibiotic prescriptions, enhancing treatment efficacy while curbing resistance proliferation. However, the scalability of XAI systems to diverse healthcare settings remains a challenge, necessitating adaptive frameworks. Integrating real‐time data streams into these models could further augment their utility in dynamic clinical environments. Future developments should aim to balance model complexity with interpretability, ensuring practical applicability in resource‐constrained settings.

The application of ML in tuberculosis (TB) resistance prediction is exemplified by Carter et al. who utilize structure‐based features to enhance pyrazinamide resistance prediction. Their gradient‐boosted decision tree model achieves notable sensitivity and specificity, identifying novel mutations for future biochemical validation. Such advancements could revolutionize TB management by enabling rapid and accurate resistance profiling. However, the variability in laboratory conditions and data quality across studies poses significant barriers to model generalizability. The integration of structural biology insights with genomic data in ML frameworks holds promise for tackling resistance in other pathogens as well (Carter et al. 2024). Pruthi et al. further highlight the potential of large‐scale WGS data in uncovering novel resistance mutations and improving predictive accuracy for multidrug‐resistant tuberculosis (MDR‐TB). By leveraging ensemble ML methods, they identify both common and rare variants associated with drug resistance, offering deeper insights into the genetic underpinnings of resistance. This comprehensive approach facilitates the identification of clinically relevant mutations and enhances the precision of resistance predictions (Pruthi et al. 2024).

These studies underline the indispensable role of exploratory approaches, data sets, and advanced algorithms in achieving clinically relevant predictions. However, challenges persist in standardizing methods across laboratories and ensuring reproducibility, which are critical for integrating these models into routine clinical practice. Moreover, the ethical considerations surrounding data privacy and algorithmic bias require addressing to ensure equitable healthcare delivery. The integration of these insights into routine diagnostic workflows requires addressing logistical and infrastructural challenges. Automated pipelines for data processing and interpretation could streamline the application of such models in clinical laboratories. Moreover, the adoption of global standards for data sharing and analysis would ensure consistent outcomes across different healthcare systems. Future research must focus on harmonizing global data repositories and refining ML techniques for widespread adoption.

## 5 Limitations

We acknowledge that the relatively small sample size (55 clinical isolates and 1 reference strain) may limit the generalizability of the findings, particularly when applying ensemble machine learning models such as Random Forest. Although the model achieved high performance metrics, further validation using larger, independent datasets is warranted to confirm the exploratory approachness of the results. Despite the limited sample size, the model achieved encouraging performance, precision, and recall—especially in CHX and TRC predictions‐likely due to strong signal in the MIC data and balanced class distribution after preprocessing.

A limitation of our study is the use of only a single reference strain (CDC MBL *C. auris* B11903) for quality control. While this strain is internationally recognized, the inclusion of multiple control strains representing different clades would further strengthen the exploratory approachness of identification and susceptibility testing. Future studies should incorporate a broader set of reference isolates to enhance comparative validity. Moreover, we selected Random Forest with restricted depth (max_depth = 5) and applied feature selection to mitigate overfitting risk commonly associated with small datasets. A limitation of our feature scaling approach is the reliance on z‐score normalization, which presumes near‐linear distribution of variables. Although log transformation and outlier inspection reduced skewness, larger datasets are needed to systematically evaluate alternative scaling methods such as exploratory approach scaling or rank transformation that may be less sensitive to non‐normal distributions. Future studies will aim to increase the number and diversity of *C. auris* isolates to train more exploratory approach and externally validated models. Cross‐institutional data sharing will be key to building a scalable and generalizable predictive framework.

The final model prioritized antifungal MIC values, which consistently demonstrated higher predictive power for biocide susceptibility. This suggests that, within the limitations of our data set, antifungal profiles may serve as surrogate markers for biocide resistance. Biocide resistance is a complex biological phenomenon potentially involving multiple molecular and environmental factors. In our study, both antifungal MIC values and phenotypic virulence factors (e.g., biofilm formation, SAP, phospholipase, esterase, hemolysin, caseinase) were initially included in the feature selection phase using the Random Forest Regressor and other embedded methods. However, none of the virulence‐related features showed significant importance scores in the model and thus were excluded from the final predictive analysis. Nevertheless, we acknowledge this as a potential limitation.

A major limitation is that the biocide resistance thresholds used here are based on limited literature and are not yet standardized across laboratories or species. Thus, while our findings provide useful comparative insights for Turkish *C. auris* isolates, the generalizability of these thresholds to other settings or clades remains uncertain. Broader inter‐laboratory studies and consensus protocols are needed to establish clinically relevant cutoffs for biocide susceptibility.

Another limitation of our study is the absence of an external validation data set. While internal validation using cross‐validation yielded strong performance metrics, external validation on independent clinical isolates is essential to confirm the generalizability of the model. Efforts are underway to mitigate overfitting risks associated with internal validation. We used conservative model parameters (e.g., max depth of 5 in decision trees) and feature scaling. Nonetheless, we agree that external validation is essential for confirming real‐world utility.

One important limitation of this study is the class imbalance in the data set, particularly for biocides such as chlorhexidine (CHX), where only three isolates were classified as resistant. This imbalance restricted the initial ability of classification models to correctly identify resistant strains. Therefore, further studies with larger and more balanced data sets are necessary to validate and enhance the generalizability of the predictive models.

## 6 Conclusion and Recommendations

The convergence of ML represents a paradigm shift in AMR research, enabling precise and actionable predictions across various pathogens. This technology offers unparalleled opportunities to enhance diagnostic accuracy, optimize treatment, and mitigate resistance emergence. However, the translation of this advancement into practice necessitates concerted efforts to address challenges such as data standardization, ethical considerations, and infrastructural limitations. Collaborative frameworks integrating researchers, clinicians, and policymakers are essential for realizing the full potential of these innovations. Furthermore, ongoing investment in research and development is imperative for staying ahead in the race against evolving resistance mechanisms. As these methodologies mature, their integration into global health strategies could significantly reduce the burden of AMR and improve patient outcomes. Developing such a predictive algorithm would provide a valuable tool for infection control and environmental decontamination strategies. Accurate predictions could reduce the need for labor‐intensive biocide testing while optimizing biocidal application. Furthermore, this approach could contribute to a deeper understanding of resistance mechanisms shared between antifungals and biocides (Hetta et al. 2023; Ahmad and Asadzadeh 2023; Pallotta et al. 2023). Future studies should aim to collect extensive susceptibility data from diverse *C. auris* isolates to train and validate these models. If successful, this strategy could enhance our ability to manage *C. auris* outbreaks effectively (Garcia‐Bustos et al. 2023). This study ultimately aims to bridge the gap in biocide susceptibility testing by leveraging the more established antifungal testing framework.

## Author Contributions

**Sidre Erganis, Ayse Seyer, Elif Ayca Sahin**, and **Ayse Kalkanci:** conceptualization, methodology, writing – original draft, writing – review and editing. **Mubarak Taiwo Mustapha, Meliz Yuvali, Abdullahi Garba Usman**, and **Dilber Uzun Ozsahin:** validation, formal analysis, visualization. **Furkan Martli, Sena Algin, Sema Turan Uzuntas, Beyza Yavuz, Alper Dogan, Esra Kılıc, Fusun Kirca:** investigation. **Cagrı Ergin and Bedia Dinc:** supervision. **Ayse Kalkanci:** funding acquisition, resources, data curation. All authors read and approved the final manuscript.

## Ethics Statement

The authors have nothing to report.

## Consent

The authors have nothing to report.

## Conflicts of Interest

The authors declare no conflicts of interest.

## Data Availability

Chromosome sequence data supporting this study's findings have been deposited in GenBank with the submission number CP147431‐ CP147486. Genbank includes 56 records associated with 8 strains, each comprising 7 chromosomes.

## References

[mbo370093-bib-0001] Ahmad, S. , and M. Asadzadeh . 2023. “Strategies to Prevent Transmission of *Candida auris* in Healthcare Settings.” Current Fungal Infection Reports 17, no. 1: 36–48. 10.1007/s12281-023-00451-7. 36718372 PMC9878498

[mbo370093-bib-0002] Angiolella, L. , F. Rojas , A. Giammarino , N. Bellucci , and G. Giusiano . 2024. “Identification of Virulence Factors in Isolates of Candida Haemulonii, *Candida albicans* and Clavispora Lusitaniae With Low Susceptibility and Resistance to Fluconazole and Amphotericin B.” Microorganisms 12, no. 1: 212. Published January 20, 2024. 10.3390/microorganisms12010212. 38276197 PMC10819056

[mbo370093-bib-0003] Ardila, C. M. , P. K. Yadalam , and D. González‐Arroyave . 2024. “Integrating Whole Genome Sequencing and Machine Learning for Predicting Antimicrobial Resistance in Critical Pathogens: A Systematic Review of Antimicrobial Susceptibility Tests.” PeerJ 12: e18213. 10.7717/peerj.18213. 39399439 PMC11470768

[mbo370093-bib-0004] Arora, P. , P. Singh , Y. Wang , et al. 2021. “Environmental Isolation of *Candida auris* From the Coastal Wetlands of Andaman Islands, India.” mBio 12, no. 2: e03181‐20. 10.1128/mBio.03181-20. 33727354 PMC8092279

[mbo370093-bib-0005] Bölükbaşı, Y. , G. Erköse Genç , G. Orhun , et al. 2021. “First Case of COVID‐19 Positive *Candida auris* Fungemia in Turkey.” Mikrobiyoloji Bulteni 55, no. 4: 648–655. 34666664 10.5578/mb.20219716

[mbo370093-bib-0006] Carter, J. J. , T. M. Walker , A. S. Walker , et al. 2024. “Prediction of Pyrazinamide Resistance in *Mycobacterium tuberculosis* Using Structure‐Based Machine‐Learning Approaches.” JAC‐Antimicrobial Resistance 6, no. 2: dlae037. 10.1093/jacamr/dlae037. 38500518 PMC10946228

[mbo370093-bib-0007] Casadevall, A. , D. P. Kontoyiannis , and V. Robert . 2021. “Environmental *Candida auris* and the Global Warming Emergence Hypothesis.” mBio 12, no. 2: e00360‐21. Published 2021 Mar 16. 10.1128/mBio.00360-21. 33727350 PMC8092241

[mbo370093-bib-0008] Cavallaro, M. , E. Moran , B. Collyer , N. D. McCarthy , C. Green , and M. J. Keeling . 2023. “Informing Antimicrobial Stewardship With Explainable AI.” PLOS Digital Health 2, no. 1: e0000162. 10.1371/journal.pdig.0000162. 36812617 PMC9931350

[mbo370093-bib-0009] Chow, N. A. , J. F. Muñoz , L. Gade , et al. 2020. “Tracing the Evolutionary History and Global Expansion of *Candida auris* Using Population Genomic Analyses.” mBio 11, no. 2: e03364‐19. 10.1128/mBio.03364-19. 32345637 PMC7188998

[mbo370093-bib-0010] Coelho, J. R. , J. A. Carriço , D. Knight , et al. 2013. “The Use of Machine Learning Methodologies to Analyse Antibiotic and Biocide Susceptibility in *Staphylococcus aureus* .” PLoS One 8, no. 2: e55582. 10.1371/journal.pone.0055582. 23431361 PMC3576404

[mbo370093-bib-0011] Cuesta, I. , C. Bielza , M. Cuenca‐Estrella , P. Larrañaga , and J. L. Rodríguez‐Tudela . 2010. “Evaluation by Data Mining Techniques of Fluconazole Breakpoints Established by the Clinical and Laboratory Standards Institute (CLSI) and Comparison With Those of the European Committee on Antimicrobial Susceptibility Testing (EUCAST).” Antimicrobial Agents and Chemotherapy 54, no. 4: 1541–1546. 10.1128/AAC.01688-09. 20124002 PMC2849363

[mbo370093-bib-0012] Çuhadar, T. , N. Karabiçak , T. Özdil , et al. 2018. “Keratit Olgularından Elde Edilen Fusarium İzolatlarının Virülans Faktörlerinin ve Antifungal Duyarlılıklarının Belirlenmesi.” Mikrobiyoloji Bulteni 52, no. 3: 247–258 (in Turkish). 10.5578/mb.66738. 30156511

[mbo370093-bib-0013] Eix, E. F. , and J. E. Nett . 2025. “ *Candidozyma auris*: Epidemiology and Antifungal Strategy.” Annual Review of Medicine 76, no. 1: 57–67. 10.1146/annurev-med-061523-021233. PMC11808652 39656947

[mbo370093-bib-0014] Erganis, S. , A. Ozturk , S. T. Uzuntas , et al. 2024. “Variable Sensitivity of Clinical *Candida auris* Strains to Biocides: Implications for Infection Control in Healthcare Settings.” BMC Microbiology 24, no. 1: 447. 10.1186/s12866-024-03605-w. 39497071 PMC11533359

[mbo370093-bib-0015] Erkose Genc, G. , I. Caklovica Kucukkaya , S. Komec , et al. 2024. “Evaluation of the First *Candidozyma auris* Isolates Reported From Türkiye in Terms of Identification by Various Methods and Susceptibility to Antifungal Drugs.” Indian Journal of Medical Microbiology 49: 100594. 10.1016/j.ijmmb.2024.100594. 38636843

[mbo370093-bib-0016] Fasciana, T. , A. Cortegiani , M. Ippolito , et al. 2020. “ *Candida auris*: An Overview of How to Screen, Detect, Test and Control This Emerging Pathogen.” Antibiotics (USSR) 9, no. 11: 778. 10.3390/antibiotics9110778. PMC7694398 33167419

[mbo370093-bib-0017] Ganeshkumar, A. , M. Muthuselvam , P. M. N. Lima , R. Rajaram , and J. C. Junqueira . 2024. “Current Perspectives of Antifungal Therapy: A Special Focus on *Candida auris* .” Journal of Fungi 10, no. 6: 408. 10.3390/jof10060408. 38921394 PMC11205254

[mbo370093-bib-0018] Garcia‐Bustos, V. , M. D. Cabañero‐Navalon , A. Ruiz‐Gaitán , M. Salavert , M. Á. Tormo‐Mas , and J. Pemán . 2023. “Climate Change, Animals, and *Candida auris*: Insights Into the Ecological Niche of a New Species From a One Health Approach.” Clinical Microbiology and Infection 29, no. 7: 858–862. 10.1016/j.cmi.2023.03.016. 36934871

[mbo370093-bib-0019] Hamilton, A. N. , F. Maes , G. Y. C. Reyes , et al. 2024. “Machine Learning and Imputation to Characterize Human Norovirus Genotype Susceptibility to Sodium Hypochlorite.” Food and Environmental Virology 16, no. 4: 492–505. 10.1007/s12560-024-09613-3. 39259473 PMC11525273

[mbo370093-bib-0020] Hayes, J. F. 2024. “ *Candida auris*: Epidemiology Update and a Review of Strategies to Prevent Spread.” Journal of Clinical Medicine 13, no. 22: 6675. Published 2024 Nov 7. 10.3390/jcm13226675. 39597821 PMC11595167

[mbo370093-bib-0021] Hetta, H. F. , Y. N. Ramadan , I. M. S. Al‐Kadmy , N. H. A. Ellah , L. Shbibe , and B. Battah . 2023. “Nanotechnology‐Based Strategies to Combat Multidrug‐Resistant *Candida auris* Infections.” Pathogens 12, no. 8: 1033. 10.3390/pathogens12081033. 37623993 PMC10458664

[mbo370093-bib-0022] Horton, M. V. , and J. E. Nett . 2020. “ *Candida auris* Infection and Biofilm Formation: Going Beyond the Surface.” Current Clinical Microbiology Reports 7, no. 3: 51–56. 10.1007/s40588-020-00143-7. 33178552 PMC7654955

[mbo370093-bib-0024] Morrissey, I. , M. R. Oggioni , D. Knight , et al. 2014. “Evaluation of Epidemiological Cut‐Off Values Indicates That Biocide Resistant Subpopulations Are Uncommon in Natural Isolates of Clinically‐Relevant Microorganisms.” PLoS One 9, no. 1: e86669. 10.1371/journal.pone.0086669. 24466194 PMC3900580

[mbo370093-bib-0025] Nwachukwu, K. C. , E. Nwarunma , C. David Uchenna , and O. Chinyere Ugbogu . 2023. “Enablers of *Candida auris* Persistence on Medical Devices and Their Mode of Eradication.” Current Medical Mycology 9, no. 1: 36–43. 10.18502/CMM.2023.150673. 37867591 PMC10590192

[mbo370093-bib-0026] Pallotta, F. , P. Viale , and F. Barchiesi . 2023. “ *Candida auris* : The New Fungal Threat.” Le infezioni in Medicina 31, no. 3 (September): 323–328. 10.53854/liim-3103-6. 37701386 PMC10495051

[mbo370093-bib-0027] Pruthi, S. S. , N. Billows , J. Thorpe , et al. 2024. “Leveraging Large‐Scale *Mycobacterium tuberculosis* Whole Genome Sequence Data to Characterise Drug‐Resistant Mutations Using Machine Learning and Statistical Approaches.” Scientific Reports 14, no. 1: 27091. 10.1038/s41598-024-77947-w. 39511309 PMC11544221

[mbo370093-bib-0028] Du Toit, A. 2023 Dec. “Sticky *Candida auris* .” Nature Reviews Microbiology 21, no. 12: 770. 10.1038/s41579-023-00986-z. 37833327

[mbo370093-bib-0029] Wang, C. C. , Y. T. Hung , C. Y. Chou , et al. 2023. “Using Random Forest to Predict Antimicrobial Minimum Inhibitory Concentrations of Nontyphoidal Salmonella in Taiwan.” Veterinary Research 54, no. 1: 11. 10.1186/s13567-023-01141-5. 36747286 PMC9903507

[mbo370093-bib-0030] Wesgate, R. , P. Grasha , and J. Y. Maillard . 2016. “Use of a Predictive Protocol to Measure the Antimicrobial Resistance Risks Associated With Biocidal Product Usage.” American Journal of Infection Control 44, no. 4: 458–464. 10.1016/j.ajic.2015.11.009. 26810885

[mbo370093-bib-0031] Zhai, B. , T. Rolling , and T. M. Hohl . 2021. “Exploring *Candida auris* in Its Habitat.” Cell Host & Microbe 29, no. 2: 150–151. 10.1016/j.chom.2021.01.010. 33571440 PMC9088162

